# Biofortification as a sustainable strategy to address micronutrient malnutrition in South Asia

**DOI:** 10.1186/s41043-025-01108-6

**Published:** 2025-12-24

**Authors:** Aneesha VijayaKumar, Irshaan Syed, Harshavardhan Dhulipalla, Payel Ghosh, Laxmikarthika V. Srinivasan, Faraz Ahmad, Sandeep Singh Rana, Vipin Singh

**Affiliations:** 1 Department of Biosciences, School of BioSciences and Technology, Vellore Institute of Technology, Vellore, 632014 India; 2 Department of Food Technology, School of Agriculture and Food Technology, Vignan’s Foundation for Science, Technology, and Research, Vadlamudi, Guntur, 522213 India; 3 Department of Integrative Biology, School of BioSciences and Technology, Vellore Institute of Technology, Vellore, 632014 India; 4 Department of Catering and Hotel Management, School of Hotel and Tourism Management, Vellore Institute of Technology, Vellore, 632014 India

**Keywords:** Malnutrition, Biofortification, Developing nations, Micronutrient deficiency, Biofortified foods, Government policies

## Abstract

Malnutrition is a significant public health concern affecting many populations, particularly in developing countries such as India and its neighboring economies. It can lead to adverse health outcomes, including stunted growth, impaired cognition, and increased susceptibility to disease. This review attempts to summarize the problems associated with malnutrition in different age groups in India and its neighboring countries, and the accompanying factors contributing to it. We also summarize the different biofortification techniques, which, when appropriately implemented, can alleviate these problems and potentially increase the availability and accessibility of crucial micronutrients such as vitamins and minerals in the diets of vulnerable populations. Government schemes and policies for scaling up biofortification interventions, as well as increasing public awareness and acceptance of biofortified crops, have also been delineated. In conclusion, while significant challenges remain, biofortification represents a promising approach for combating malnutrition, although further research and evaluations are needed to realize its potential impact fully.

## Introduction

 Malnutrition remains one of the most persistent global public health challenges, despite notable progress in agriculture, healthcare, and socioeconomic development [1]. Its impact is particularly severe in South Asia, especially in India, Pakistan, Nepal, Bangladesh, Sri Lanka, and parts of China, where demographic pressures, economic disparities, and environmental stressors converge to exacerbate nutritional deficits [2]. In these countries, poverty and high food prices remain primary drivers, restricting access to diverse, nutrient-rich diets. The situation is further compounded by climate change, which disrupts crop cycles, reduces yield, and leads to seasonal food insecurity [3]. 

Micronutrient deficiencies are widespread, with iron, zinc, calcium, vitamin B12, and vitamin D shortages being especially common [4]. These deficiencies affect not only vulnerable groups such as children and pregnant women but also working-age adults and the elderly, resulting in diminished productivity and increased healthcare burden. The magnitude of the crisis is reflected in the statistic that India alone accounts for nearly one-third of the world’s malnourished children [5]. 

The global nutritional landscape is now shaped by a triple burden. Undernutrition includes stunting, wasting, and underweight, which remain prevalent in low-income and rural areas [6]. According to the 2025 estimates, 150.2 million children under five are stunted, and 42.8 million are wasted worldwide. These conditions impair cognitive and physical development, perpetuating cycles of poor educational attainment, reduced earning capacity, and increased susceptibility to illness [7]. 

Micronutrient deficiencies are characterized by insufficient intake of vitamins and minerals, even in populations meeting caloric needs. Deficiencies in iron, zinc, and vitamin A alone affect billions of people globally, contributing to anemia, impaired immunity, developmental delays, and higher mortality rates. The problem often goes unnoticed until irreversible health impacts occur [8]. 

Overnutrition consists of overweight and obesity. It is rising rapidly, even in traditionally undernourished regions, driven by urbanization, dietary shifts towards processed foods, and sedentary lifestyles. In 2022, 15.8% of adults globally were obese, with that figure projected to surpass 1.2 billion by 2030. This trend is associated with an increased prevalence of non-communicable diseases such as type 2 diabetes, hypertension, and cardiovascular illnesses [9]. 

Addressing all three forms of malnutrition requires a multi-pronged approach. Traditional interventions such as supplementation and industrial fortification have achieved measurable success but often face sustainability and accessibility challenges, particularly in rural and low-income communities. Biofortification, defined as the process of increasing the bioavailable micronutrient content of staple crops through agronomic methods, conventional breeding, or modern biotechnological techniques (e.g., genetic engineering, genome editing), presents a cost-effective, long-term strategy for improving nutritional outcomes in populations with limited dietary diversity [10]. 

Unlike post-harvest fortification, biofortification builds nutrition directly into the crop during production, ensuring that the benefits reach even those outside organized food distribution systems. Zinc-enriched rice varieties in India and Bangladesh, shown to improve plasma zinc levels in children. Iron-rich pearl millet in Maharashtra, India, reduces anemia prevalence in school-aged children. Provitamin A sweet potato in African countries lowers vitamin A deficiency rates [11]. 

The promise of biofortification has been recognized at the highest policy levels. In 2023, the World Health Assembly passed a resolution urging member states to expand national biofortification programs, integrate them into agricultural policy, and link them with public health initiatives [12]. This review aims to provide a comprehensive, evidence-based synthesis of malnutrition and biofortification in the South Asian context. Specifically, it will access the prevalence, trends, and underlying drivers of malnutrition in India and its neighbouring countries, incorporating the most recent (2023–2024) region-specific data. Evaluate biofortification techniques such as agronomic, conventional breeding, and transgenic/genome editing approaches with emphasis on their technical feasibility, scalability, and applicability in South Asian agricultural systems. Analyze existing biofortified crop varieties in rice, wheat, millets, pulses, and other staples, including health impact data, adoption trends, and socio-economic barriers. Examine national and regional government schemes, policies, and public-private initiatives aimed at promoting biofortification, identifying gaps between policy intent and field-level implementation. Highlight future research and policy priorities, including integration with climate-smart agriculture, farmer incentives, and consumer acceptance strategies.

By integrating epidemiological insights with agronomic and policy perspectives, this review positions biofortification not a a standalone solution, but as a core pillar in the comprehensive nutrition strategy capable of complementing supplementation, biofortification, and dietary diversification efforts. The novelty of this work lies in its country-specific analysis, its emphasis on comparative evaluation of biofortification approaches, and its critical linkage between agricultural innovation and public health outcomes in the South Asian setting.

## Country-wise malnutrition scenario

### India

#### Prevalence

India faces enduring malnutrition across all age groups, despite improvements over the past decades [3]. According to the 2024 Global Hunger Index, India scores 27.3, which remains “serious.“ [13] Recent NFHS-5 and government statistics suggest that about 35.5–38.9% of children under five are stunted, 17–19.3% are wasted, and around 32% are underweight; 67% of children aged 6–59 months are anemic, marking an increase over the previous years [14, 15]. For women aged 15–49, anemia prevalence is about 57%. Under-nourishment is also widespread among adults, especially among marginalized communities. The underlying food insecurity is also chronic—over 74% of the population cannot afford a healthy diet, and a significant portion lags behind nutrient-adequate diets [16]. 

Child malnutrition in India is a public health concern and a serious impediment to the development of the country [17]. Evidence from research in underdeveloped countries indicates that increased education is very much linked with a significant decline in the rate of underweight births, from 43% to 15.5% [18]. Malnutrition in children under the age of five was the primary risk factor that impacted adolescents in all Indian states in 2017. A child’s nutritional status acts as a critical determinant of their adult health and life course [19]. Child malnutrition in India shows large regional variations: underweight prevalence among children younger than five varies between 39% and 75%, stunting from 15.4% to 74%, and wasting from 10.6% to 42.3%. Large gender and rural–urban disparities also add to these variations, with girls and rural children having consistently worse growth and nutrition status compared with boys and their urban peers [20].

Malnutrition in India continues to be alarmingly high in relative and absolute terms. Adult malnutrition remains a major public health concern, a sign of entrenched nutritional disparities. Adolescent girls are particularly at risk, with iron deficiency anemia common, given higher nutritional needs during adolescence and menstrual blood loss [21]. 

While the magnitude of malnutrition in India is well established, its persistence cannot be explained by prevalence data alone. To understand why such high levels persist despite economic progress, it is essential to examine the underlying factors that contribute to this nutritional crisis.

#### Contributing factors

The persistence of malnutrition in India is driven by an interplay of poverty, poor maternal health, gender discrimination, and limited dietary diversity [22]. Insufficient maternal nutrition and education lead to poor early childhood outcomes [23]. Environmental factors, such as inadequate sanitation and unsafe water, contribute to frequent gastrointestinal diseases, which impair nutrient absorption. Large regional, rural-urban, and gender disparities exist; girls and children from rural or marginalized backgrounds face added risk. Inadequate coverage, inefficient targeting, and implementation lapses in government nutrition programs compound the challenge [18]. 

These risk factors, ranging from poor maternal health to sanitation gaps, do not just explain why malnutrition remains entrenched; they also directly translate into severe health and developmental outcomes. The consequences of malnutrition and micronutrient deficiencies in India highlight the urgency of addressing these drivers through targeted interventions.

#### Consequences of malnutrition and micronutrient deficiencies in India

Malnutrition in India causes general health and social problems, such as high child mortality, stunted cognitive and physical growth, and reduced economic productivity. Early stunting, anemia, and wasting have long-term impacts on educational performance and life-long health. In adults, particularly women and the elderly population, malnutrition leads to chronic illnesses, frequent illnesses, and lower life expectancy. These trends perpetuate intergenerational cycles of poverty and poor health, ultimately affecting national development.

Deficiency of Vitamin D: 61%.

Iron deficiency: 54%.

Deficiency of Vitamin B₁₂: 53%.

Deficiency of folic acid: 37%.

Iodine deficiency: 17%.

Deficiency of Vitamin A: 19% [24].

The effects of micronutrient deficiencies are extensive and severe. They affect physical and mental development in children, lower productivity in adults, and enhance vulnerability to infections and diseases. In pregnant women, deficiencies can result in poor birth outcomes, such as low birth weight and neonatal mortality. Overcoming these issues requires a holistic approach. Though India has instituted numerous programs targeting malnutrition, such as supplementation and food biofortification programs, the continued prevalence of high rates of deficiency is an indication of the necessity of more efficient interventions. Combining nutrition education, enhancing dietary variety, and maximizing the coverage and quality of available programs are some key steps to reducing the impact of malnutrition in the nation [24]. 

### Pakistan

#### Prevalence

Pakistan is still among the worst-affected nations globally regarding undernutrition [25]. Over 40% of children under the age of five are stunted, 17.7% wasted, and 28.9% underweight, based on figures up to 2024. Anemia levels in children and women exceed 50% and 42%, respectively. Latest statistics indicate millions of children and women are acutely food insecure, with an estimated 1.4 million infants born into hunger in 2024 alone. Regional and gender gaps are large, and crises such as climate shocks are particularly dangerous [26].

Stunting and child malnutrition are severe health problems in South Asia, East Africa, and West Africa, with the majority of stunted children (around 57.9 million) living in South Asia [27]. In Pakistan, malnutrition contributes to annual economic losses of approximately US$7.6 billion due to poor early childhood growth and development [27]. Malnutrition affects all age groups, particularly newborns, young children, teenagers, pregnant women, and nursing mothers.

Malnutrition in Pakistan is exacerbated by climate change, economic instability, and poor healthcare infrastructure. These challenges demand multi-pronged policies aimed at improving agricultural productivity, strengthening healthcare systems, and expanding social safety nets to ensure food and nutrition security for all. Pakistan continues to experience a heavy malnutrition burden, and its rapid population growth risks worsening the crisis [28].

While these statistics highlight the scale of Pakistan’s malnutrition burden, understanding why these numbers remain so persistently high requires an exploration of the contributing factors that sustain the crisis.

#### Contributing factors

Main drivers are extensive poverty, long-term food insecurity, poor maternal health, and poor infant feeding practices [29]. Only 38% of children are breastfed exclusively up to their first six months. Floods and droughts, which are climate shocks, affect food systems and livelihoods, raising vulnerability even more. Structural deficits in health and protection systems, combined with high prices for food and large, dependent household sizes, perpetuate high malnutrition. Shortages of key vitamins and minerals, including iron, zinc, vitamin A, and D, continue to be prevalent in the diet [29, 30].

These interlinked drivers, poverty, food insecurity, inadequate healthcare, and climate shocks, not only explain the persistence of malnutrition but also directly translate into grave consequences for health, productivity, and national development.

#### Consequences of malnutrition

Malnutrition leads to high rates of child death, slow growth, and cognitive impairment, as well as increased deaths among mothers and newborns [31]. This costs the economy more than US$17 billion a year. The crisis makes people poorer, makes workers less productive, and puts a huge load on the health care system. As overweight and obesity rates rise, Pakistan faces a twofold burden, particularly in urban and affluent areas, increasing risks for all of society [32].

### China

#### Prevalence

China’s nutrition profile has dramatically altered, with consistent improvement in decreasing undernutrition but new threats from overnutrition [33]. In 2024, stunting in children under five is approximately 4.2–4.8%, and wasting is 1.6–1.9% [34]. Yet, overweight in the under-fives is 8.5–8.9%, a manifestation of the double burden of malnutrition. The proportion of the population that is undernourished is now less than 2.5%, but micronutrient deficiencies and rural-urban inequalities continue to exist. Anemia is still 15.5% in women of reproductive age [35].

Approximately 150.8 million individuals are malnourished. High rates of 25% obesity, 19.6% anemia, and 9.4% stunting in youngsters between 18 and 25 present are serious national and worldwide concerns, given the size of China’s population. China and other middle-income countries are not immune to the numerous consequences of undernutrition from insufficient protein, energy, and other vital nutrients. The dietary challenges faced by the poor in middle-income countries are comparable to those faced by their counterparts in less industrialized countries [36]. China’s nutritional adequacy regarding accessible food energy has dramatically improved in recent decades due to the government prioritizing food security [37]. 

Although these statistics capture the shifting nutritional landscape in China, they do not explain why malnutrition, both undernutrition and overnutrition, remains persistent. To understand this, it is necessary to examine the contributing factors underlying these trends.

#### Contributing factors

Chronic rural poverty, insufficient healthcare access, and significant alterations in food systems are among the primary culprits [38]. Infrastructure and markets are challenging for smallholder farmers in far-off regions to deal with [39]. This makes it tougher for people to receive food and limits the kinds of food they can get. More people are eating foods that are high in calories and lacking in micronutrients because cities are growing and people’s diets are changing. This has made people more likely to have chronic diseases and become obese, especially in cities. Micronutrient deficiencies and functional impairments are more prevalent in older and rural populations [40].

These demographic, health-related, and dietary determinants do not exist in isolation. They translate directly into serious health outcomes across the life course. The consequences of malnutrition in China reveal the profound long-term impact it has on individuals and society.

#### Consequences of malnutrition

China faces a double burden: while fewer children are stunted or wasted, the rates of childhood overweight, adult obesity, and associated non-communicable diseases are climbing [41]. In younger children, undernutrition still causes morbidity and affects lifelong health and learning [42]. Among adults, especially the elderly, malnutrition accelerates frailty, increases chronic disease, and challenges healthy aging. These issues are particularly acute in rural and low-income populations. Nationally, the population health burden is shifting from infectious to chronic, nutrition-related diseases [34].

### Bangladesh

#### Prevalence

Bangladesh has achieved impressive reductions in overall child malnutrition; however, it still experiences high levels compared to regional and international standards [43]. The 2024 Global Hunger Index rates Bangladesh 19.4, as a “moderate” level of hunger. The latest BDHS 2022 statistics indicate that 24–28% of children under the age of five are stunted, 22% are underweight, and around 10–11% are wasted. Exclusive breastfeeding rates have increased to more than 62%, but almost 27.8% of infants are born with low birth weights [44]. Over 36% of reproductive-aged women suffer from anemia, and micronutrient malnutrition is prevalent among the population. Adult obesity levels are low (6–7% for women), but the double burden of malnutrition is becoming a growing issue, particularly in urban regions [45].

According to the WHO (2020) data, Bangladesh is one of the top ten Southeast Asian nations with the highest rate of under-five child fatalities. According to the Bangladesh Demographic and Health Survey 2007, 43% and 41% of Bangladesh’s youngsters were stunted and underweight [46]. A recent study of child malnutrition in Bangladesh found that stunting levels ranged from 28% to 51% at the sub-district level and from 34% to 48% at the district level [47]. Multivariate analysis revealed that children of highly educated mothers and families with higher income indices had a lower prevalence of malnutrition than the reference group [48]. Poverty rates in Bangladesh remain high, and poverty must be decreased, which aims to reduce child malnutrition in these areas because economic status and child nutrition are negatively associated.

While these highlight the widespread burden of malnutrition across different age groups, the persistence of such high rates can only be fully understood by examining the contributing factors that drive these outcomes.

#### Contributing factors

The primary causes of malnutrition in Bangladesh include multi-dimensional poverty, limited levels of maternal education, and insufficient food, primarily in rural communities. The highest levels of malnutrition are seen among rural households, children of women who are illiterate, and the lowest fifth of the population [47]. Access to sanitation and clean water has improved, but it is unevenly distributed, particularly in marginal or flood-prone communities. All of these are worsened by gender inequality, poor feeding practices among infants and young children, and fluctuating food prices due to natural shocks. Most of the families lack diversity in their diets due to overconsumption of rice and a lack of sufficient fruits, vegetables, and animal-source foods [48].

These determinants, spanning social, economic, and health-related domains, create conditions that perpetuate poor nutritional status throughout the life cycle. Their impact is visible in the consequences of malnutrition, which extend from childhood into adulthood and even across generations.

#### Consequences of malnutrition in Bangladesh

Severe malnutrition over the long term increases death rates and damages physical and mental development, eventually reducing future human capital. Underweight and short children are more susceptible to illness and poor performance at school. Chronically undernourished and anaemic women are more likely to bear low birth weight babies and other complications during pregnancy, which creates a cycle of starvation. The increase in overweight and obesity among children and adolescents, particularly in urban centers, adds to the risks of non-communicable diseases, adding to which already prevalent issue of undernutrition [49].

### Nepal

#### Prevalence

Nepal has made considerable gains in the reduction of stunting, yet rates of malnutrition are high, particularly in certain provinces and rural settings [50]. In 2024, about 25–32% of children under age five are stunted, 12% wasted, and 19% underweight. More than one-third of women of reproductive age are anemic, and 21.8% of newborns are low birth weight [51]. Despite advances, the rates of exclusive breastfeeding have plateaued at approximately 62%, and one out of four Nepali children is still stunted. Overweight among children (2.6%) and adults is a new trend, and rates of obesity and diabetes continue to increase, especially among urban and affluent groups [50].

According to surveys, 14.5–17% of Nepalese adult women are underweight [52]. In Nepal, there were 2.2 million old people in 2011 compared to 1.5 million in 2001, as reported by the National Census. 85% of elderly persons in Nepal live in rural areas [53]. The cumulative effect of several variables, such as physical inactivity, lack of appetite, various ailments, and severe economic conditions, can all contribute to geriatric malnutrition.

Low weight-for-age prevalence likewise exhibits a sharp increase from 6 to 18 months and subsequent flattening [51]. Despite pervasive discrimination against girls, caste distinctions, and untouchability in Nepal, there is little correlation between gender, ethnicity, and malnutrition [53]. 

#### Contributing factors

Principal drivers are chronic poverty—approximately 20% of the population is below the poverty line—climate-induced food insecurity, as well as sweeping regional and social inequalities. Malnutrition due to micronutrient deficiency remains extremely high, as do incidence rates for women not having access to adequate healthcare and education, particularly in rural or mountain regions [51]. Sanitation and water improvements have not matched urban growth, and there are disparities between males and females, although these are lower than in some surrounding countries. Poor newborn feeding behavior and dietary insufficiency are the causes of the burden of malnutrition due to stunting and wasting, particularly in the Terai and Madhesh regions [53].

#### Consequences of malnutrition in Nepal

The ramifications are serious: long-term undernutrition of children hampers mental and physical growth, makes them more vulnerable to illness, and restricts educational and economic success [54]. Undernutrition in mothers and adolescents creates a vicious cycle of adverse pregnancy outcomes and infant illness [55]. Childhood undernutrition is a primary driver of Nepal’s under-five mortality and of the national disease burden, whereas overweight and obesity are starting to bring long-term threats for non-communicable diseases [56].

### Sri Lanka

#### Prevalence

Sri Lanka was once a frontrunner in human development, but now experiences increasing malnutrition due to chronic economic issues [57]. Sri Lanka ranks 11.3 on the 2024 Global Hunger Index and has a “moderate” hunger level. It ranks 56 among 127 nations. Based on national surveys carried out recently, 12 to 17% of children under five are stunted, 10 to 15% are underweight, and 10 to 11% are wasted [58]. Nearly a third of children under five are not being well fed. This is particularly prevalent in rural and estate areas. Over 26% of the population will be below the poverty line in 2024. Currently, approximately 43% of families make use of food coping strategies, like skipping a meal, eating less, or consuming more consumption of foods that they do not enjoy. Over 40% of females aged 18 to 60 years are overweight and obese since they do not consume a variety of meals. This is evidently a double burden [57].

Stunting, wasting, being overweight, and obesity are common among children aged 6 to 16. This population’s presence of overweight/obese, high prevalence of wasting, and low frequency of stunting suggests public health issues and a double burden of malnutrition [57]. 

#### Contributing factors

The growth in malnutrition is caused by a number of things that are all connected: an economic crisis, rising food prices, falling salaries, and natural calamities that happen every few years. Urbanization has resulted in more people eating inexpensive, low-nutrient meals, which has hurt rural, estate, and vulnerable communities the most. Pregnant women don’t eat enough, babies don’t get enough protein and micronutrients, and it’s still hard to find foods that are fortified with these nutrients. This is especially true in the north, east, and estate regions. The situation is made worse by gender inequality, a lack of knowledge about nutrition, and weak social safety nets [58].

#### Consequences of malnutrition in Sri Lanka

Malnutrition has contributed to heightened under-five mortality, suboptimal child growth and development, and mounting health hazards for both the under- and over-nourished. Wasted and stunted children exhibit poor educational achievements and earning potential [59]. Adults, particularly women, with overnutrition have increased incidences of diabetes and cardiovascular complications. Malnutrition’s health and economic burdens are on the rise, burdening public healthcare and stifling Sri Lanka’s journey towards recovery and sustainable development [60].

Table 1 shows the different factors contributing to malnutrition and its associated health outcomes across physiological groups in South Asian countries. Malnutrition in South Asian nations is a result of several interrelated issues that affect various groups, such as infants, children, adolescents, pregnant women, and the elderly. Poverty stands out as a primary contributor, which does not enable families to obtain appropriate nutrition and access to quality healthcare. Malnutrition in pregnant women may result in weak or undernourished babies. Most people also do not know as much as they should about good food, breastfeeding, and cleanliness, which contributes to the problem. Girls and women tend to eat less than others in the family, which increases their likelihood of being malnourished. Poor sanitation and contaminated water lead to many diseases that prevent the body from benefiting from the food. In a few areas, cultural beliefs and food regulations keep people from consuming some healthy foods. All these problems combined form a cycle of malnutrition that is difficult to escape [28]. 

**Table 1:** Factors contributing to malnutrition across physiological groups in South Asian countries

S. No	Country	Physiological group	Factors contributing to malnutrition	Associated health outcome	Reference
1.	India	Children under 5	Poor feeding, WASH, maternal undernutrition	Stunting (35.5%), wasting, and mortality	[61]
		Adolescent	Iron deficiency, early marriage, and poor diet	Anemia (56%), and low birth weight	[62]
		Pregnant and lactating women	Micronutrient deficiencies, low pregnancy weight	Maternal anemia (75%), low birth weight infants	[63]
		Elderly	Illness, poor diet, food insecurity	Frailty, cognitive decline, and underweight	[64]
2.	Pakistan	Children under 5	Poverty, healthcare access, and maternal diet	Stunting (40.2%), wasting	[25]
		Adolescent girls	Micronutrient & gender disparities	Anemia, dropouts, pregnancy risks	[65]
		Pregnant and lactating women	Low BMI, poor ANC access, vitamin A, and Iron deficiency	Maternal/infant mortality	[66]
		Elderly	Undernutrition, lack of social support, and chronic illness	Underweight, depression, reduced immunity	[67]
3.	China	Children under 5	Regional/rural feeding deficits	Stunting (5.8%), increasing overweight	[68]
		Adolescent girls	Changing diet, iron/calcium lack	Anemia, obesity risk	[69]
		Pregnant and lactating women	Poor diet, folic acid, and iodine lack	Birth defects, low birth weight	[70]
		Elderly	Isolation, multimorbidity, nutrient deficiencies	Sarcopenia, micronutrient deficiencies	[71]
4.	Bangladesh	Children under 5	Food insecurity, poor sanitation, and maternal anemia	Stunting (31%), and wasting (8.4%)	[72]
		Adolescent girls	Low iron food, early marriage	Anemia (50%), early pregnancy risks	[73]
		Pregnant and lactating women	High anemia rates, inadequate weight gain, and poor diet	Low birth weight, maternal complications	[73]
		Elderly	Neglect, poverty, chronic conditions	Underweight, fatigue, and poor immunity	[74]
5.	Nepal	Children under 5	Poor food diversity, hygiene, maternal education		
		Adolescent girls	Iron deficiency, poverty, and early marriage	Anemia, reduced school performance	[75]
		Pregnant and lactating women	Low BMI, poor ANC, iron deficit	Low birth weight, maternal mortality	[76]
		Elderly	Poor diet, diseases	Nutrient deficiency, reduced functional capacity	[77]
6.	Sri Lanka	Children under 5	Regional disparities, poor nutrition education	Underweight, anemia	[28]
		Adolescent girls	Body image, micronutrient deficit, and a bad diet	Thinness (25%), anemia, and stunted growth	[78]
		Pregnant and lactating women	Iron and calcium deficiencies, low dietary diversity	Anemia (75%), pregnancy complications	[79]
		Elderly	Poor food access, disease, and malabsorption	Frailty, underweight (12.5%)	[80]

## Biofortification techniques

Biofortification is increasingly recognized as an affordable, scalable, and environmentally sustainable strategy to combat micronutrient deficiencies. By enhancing the nutrient content of staple crops through agronomic practices, traditional breeding, or genetic approaches, biofortification directly targets the diets of vulnerable populations. Successful examples include iron-enriched pearl millet, zinc-fortified rice, and vitamin A-biofortified sweet potato, which have demonstrated measurable impacts on both health and economic outcomes [27]. To maximize its potential, biofortification must move beyond research and pilot programs into mainstream agricultural policy, farmer adoption, and consumer awareness initiatives that address hidden hunger on a larger scale [81].

### Agronomic techniques

Agronomic approaches enhance the nutrient content of crops primarily through soil and fertilizer management. This includes applying micronutrient-enriched fertilizers, optimizing soil fertility, and using foliar sprays to boost nutrient uptake and accumulation in edible plant parts [82]. By improving nutrient availability, plant uptake efficiency, and microbial activity, agronomic biofortification offers a relatively low-cost and straightforward solution. However, its effectiveness depends heavily on environmental conditions, crop type, and correct application practices [83]. 

Agronomic biofortification has shown promise in addressing South Asia’s high burden of malnutrition, particularly in countries such as India, Bangladesh, Nepal, and Pakistan [84]. For instance, zinc-biofortified rice in Bangladesh and iron-biofortified pearl millet in India have contributed to improvements in dietary intake and reduced prevalence of iron-deficiency anemia and zinc deficiency. When integrated into local cropping systems and supported by agricultural policy, these approaches demonstrate substantial public health benefits [85, 86].

Yet, scaling agronomic interventions requires more than technical feasibility. Their success hinges on institutional support, farmer adoption, market access, and public awareness. Evidence suggests that countries with stronger institutional backing for agronomic biofortification experience more consistent improvements in nutritional outcomes [87]. 

#### Nutrient seed priming

**Fig. 1:**
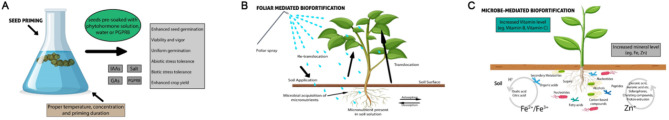
Seed Priming (**A**), Foliar-mediated (**B**), and Microbe-mediated (**C**) biofortification methods

Within agronomic techniques, seed priming has emerged as a simple yet revolutionary practice (Fig. 1A). This involves treating seeds with biological agents ot nutrient solutions before sowing, thereby enhancing their physiological performance. Seed priming improves soil fertility, stress tolerance, and nutrient uptake, restoring agroecological balance and strengthening crop resilience [88]. The activation of metabolic processes during pre-germination promotes growth and equips the plant with adaptive stress responses, ultimately leading to higher yields and improved nutritional quality [89]. 

Numerous physicochemical parameters, including aeration, seed quality, osmotic and water potential, priming agent, time, temperature, and the presence or absence of light, can influence seed germination. Therefore, various techniques for seed priming exist, including hydro priming, halo-priming, osmo-priming, biopriming, chemical priming, and hormonal priming. Seed priming is easy, inexpensive, and can be performed just before sowing by small-scale farmers. Moreover, the durability of its effects after drying allows for integration with industrial seed dressing processes [90]. Experimental studies further highlight its benefits. For instance, Alam et al [91] simulated drought conditions in wheat and demonstrated that priming with salicylic acid, ascorbic acid, and NaCl improved germination and drought resistance by upregulating antioxidant defense and glyoxalase systems. Similarly, Rasooli et al. [92] showed that Cold Plasma (CP) priming enhanced cumin seedling development, root morphology, antioxidant activity, and nutrient homeostasis, suggesting its potential in aromatic and medicinal crops.

While seed priming provides an efficient and low-cost solution for improving germination and early vigor, its effects are often restricted to the initial growth stages. To ensure sustained nutrient enrichment during later stages of crop development, foliar and flood-mediated biofortification techniques have emerged as valuable alternatives.

#### Foliar and flood-mediated biofortification

One of the most effective methods to enhance Se, I, Cu, Fe, Zn, and, to a lesser extent, Mg levels in cereal grains is foliar mineral application (Fig. 1B). Evidence accumulated over two decades suggests that foliar spraying can increase crop yield and mineral density, though the effects vary under different environmental conditions. In wheat, rice, and maize, foliar treatments have been reported to elevate mineral densities up to 98, 69, and 30-fold for I; 23, 5, and 134-fold for Se; 444%, 311%, and 122% for Zn; and 73%, 87%, and 107% for Fe compared with controls.

Foliar feeding has proven to be 15–19% more efficient than soil application since nutrients are delivered directly to metabolically active leaves, resulting in quicker uptake [93] and [94]. In most cases, foliar application close to crop maturity complements soil application at sowing, contributing more to yield and grain quality, respectively. For perennial crops such as citrus, where soil micronutrient uptake may be limited due to deep roots, foliar sprays have shown clear yield and fruit quality benefits [95]. Importantly, only a fraction of the fertilizer required for soil application is needed when applied as a foliar spray, making it a more profitable option.

Although foliar and flood-mediated applications deliver rapid improvements in crop yield and nutrient concentration, they depend on repeated external inputs and favorable field conditions. To reduce reliance on chemical fertilizers and leverage natural processes, researchers have turned to microbe-mediated biofortification.

#### Microbe-mediated biofortification

An increasingly promising approach is the use of microorganisms with nutrient-sequestering abilities and plant growth-promoting traits (Fig. 1C). These microbes enhance nutrient solubilization in the rhizophore and facilitate translocation into edible plant parts [96]. A literature review indicates that bacteria that promote plant growth may be effective bio-fortifiers of micronutrients in grains [97]. One might achieve biofortification through microbes by utilizing different microbial biofertilizers, which solubilize the critical minerals and micronutrients in soil and make them readily available for plants to absorb [98]. 

For example, Gupta et al. [99] demonstrated that arbuscular mycorrhizal fungi reduced As accumulation while simultaneously enhancing Se, Ni, Mn, Zn, Fe, Ca, Mg, K, N, and P concentrations in wheat grains, a strategy particularly valuable in As-contaminated regions. Similarly, Singh et al. [100] found that co-inoculation of *Azotobacter chroococcum*, a rhizobacterium, and *Piriformospora indica*, a photo-promoting fungal endophyte, improved zinc (Zn) and iron (Fe) uptake in wheat through altered root exudates, enhanced low-molecular-weight organic acid profiles, and upregulated transporter gene expression. 

While agronomic biofortification can deliver quick nutrient gains, these improvements are often temporary and dependent on continuous input application. To achieve permanent, self-sustaining increases in crop nutrient density, scientists have turned to breeding approaches that embed these traits into the plant’s genetic makeup.

### Traditional breeding techniques

**Fig. 2:**
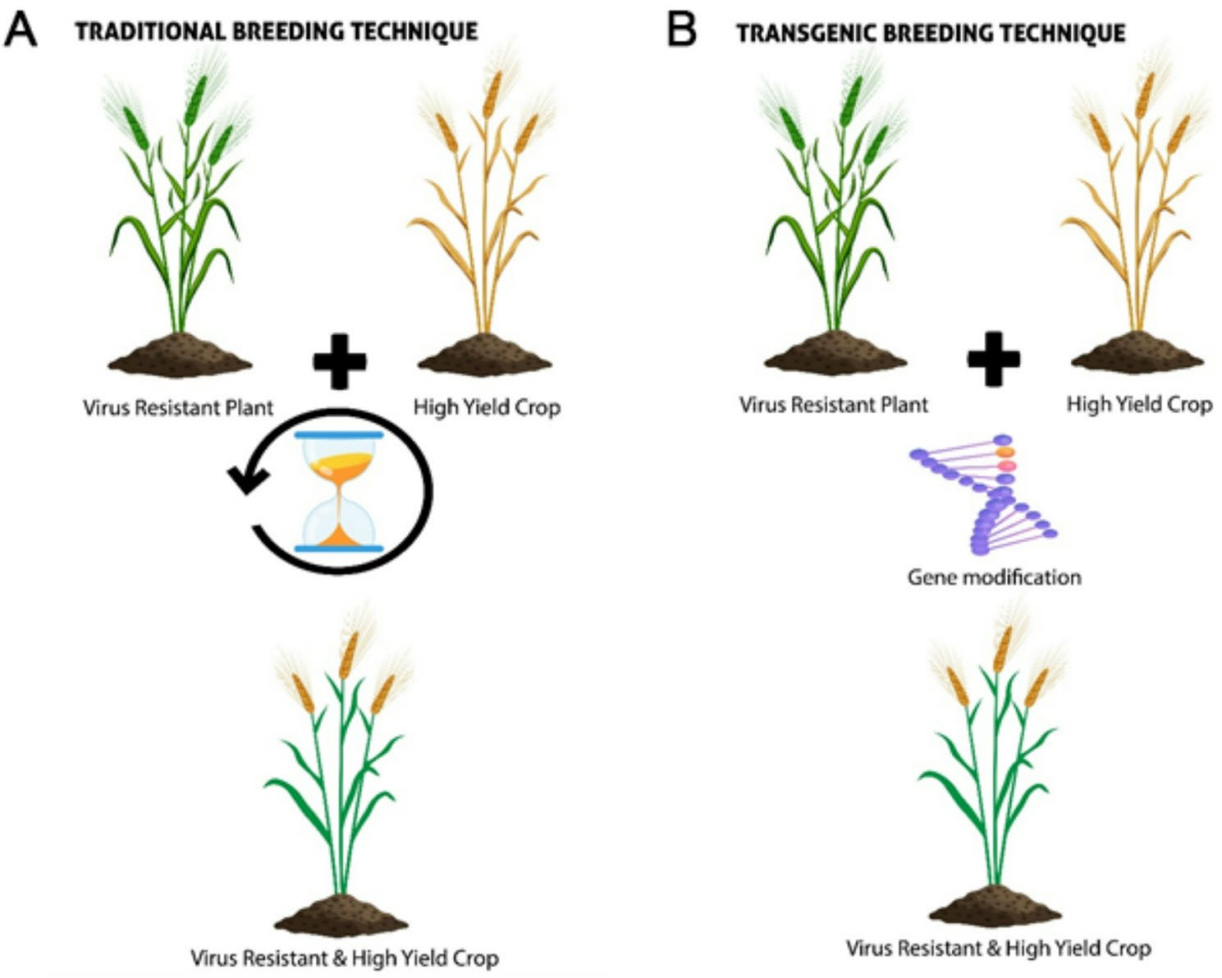
Traditional (**A**) and Transgenic (**B**) breeding techniques

The time-tested practice of conventional breeding (Fig. 2A) has evolved with scientific and technological advances. It primarily exploits inherent genetic variability within a species and, in some cases, among closely related taxa that are not reproductively isolated [101]. Through controlled crossing, breeders combine desirable traits from multiple parents into improved cultivars [102]. 

Consumers generally expect foods derived from conventional crops to be safe and nutritious, although few have been formally tested for potential risks. Traditional breeding through self or cross pollination, shuffles allelic combinations without introducing novel biochemical pathways. Breeders can tailor strategies to individual crops while accounting for natural toxins unique to each species [103]. 

Traditional breeding has successfully generated nutrient-enriched crop varieties. However, its effectiveness is limited by the genetic variation available in the crop’s gene pool. Desired micronutrient traits may not always be present, or may be linked to undesirable agronomic characteristics, slowing progress. These limitations have driven the development of alternative molecular approaches. To overcome these genetic barriers and accelerate improvement, transgenic techniques were introduced, enabling direct manipulation of plant genomes.

### Transgenic techniques

Transgenic breeding represents a powerful alternative to traditional breeding, offering the ability to transfer or modify genes with high precision (Fig. 2B). Over the last two decades, advances in genetic engineering have enabled targeted introduction of beneficial traits, particularly those enhancing crop performance under abiotic stress [104]. Within transgenic methods, genome editing technologies, especially CRISPR, have emerged as the most transformative tool for biofortification.

#### CRISPR-mediated biofortification

**Fig. 3:**
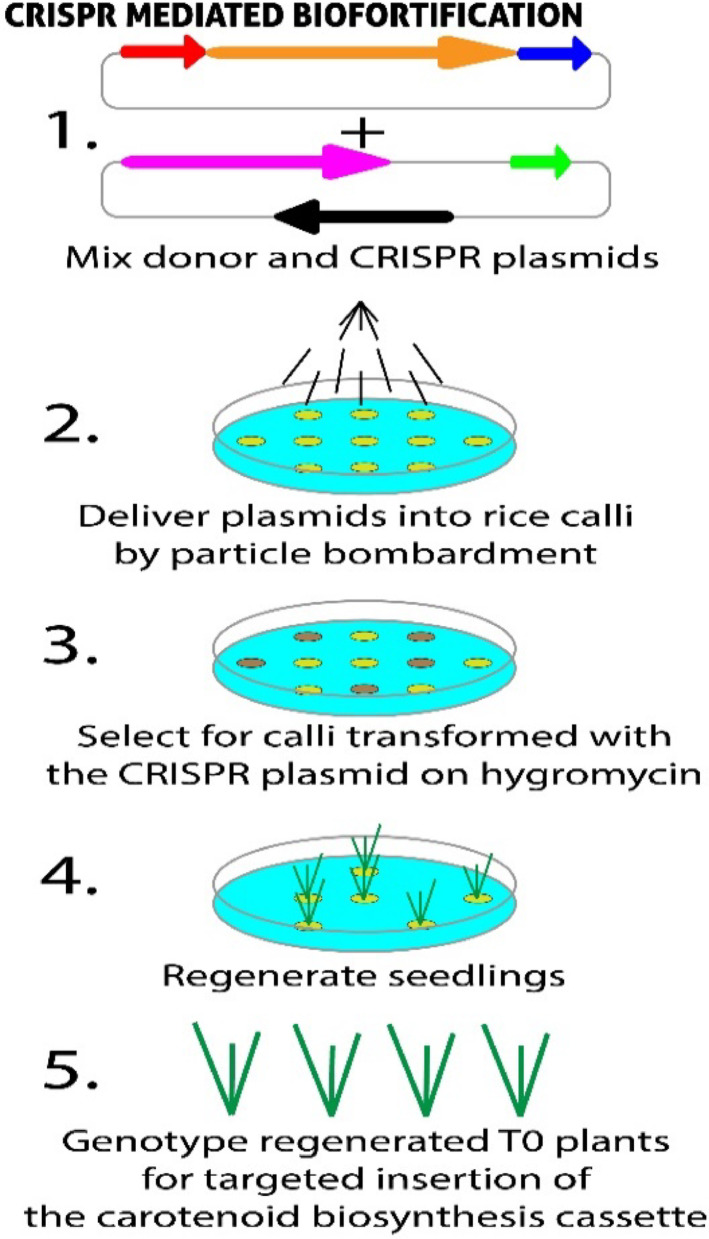
CRISPR/Cas9 technology-based biofortification

CSISPR/Cas systems have revolutionized plant genome editing, allowing precise modification of genes involved in nutrient biosynthesis, accumulation, and storage. This approach has been successfully applied to improve micronutrient density in rice, tomato, and maize, particularly for iron, zinc, and provitamin A [105]. (Fig. 3). Beyond nutrition, CRISPR has also enhanced tolerance to drought and salinity, helping secure yield stability under climate stress [106]. such dual improvements make CRISPR-Cas9 technology has proved to be a potent genome editing tool for biofortifying crops through the accurate modification of genes that participate in nutrient biosynthesis, accumulation, and storage. Research like that of Chakravorty et al. [105] shows how CRISPR can improve the nutritional value and shelf life of crops by modifying genes responsible for micronutrient biosynthesis (e.g., iron, zinc, and provitamin A). These specific interventions have demonstrated promising results in elevating the nutrient content of foods such as rice, tomato, and maize, providing a direct route to the fight against hidden hunger and micronutrient malnutrition. 

Moreover, Shelake et al. [106] show that CRISPR has also been used to enhance crop tolerance to abiotic stresses such as drought and salinity, thereby indirectly contributing to food and nutrition security by keeping yields stable under stress conditions. This double effect—increasing both nutritional quality and agronomic yield—is rendering CRISPR an extremely strategic weapon in the war against malnutrition, particularly in climate-exposed areas. 

Nonetheless, there are problems. Although highly specific, CRISPR biofortification suffers from regulatory hurdles, off-targeting, and social acceptance concerns, especially among countries with tightly regulated GMO guidelines. In addition, most improvements derived from CRISPR currently remain in an experimental phase and have had fewer applications in large-scale food systems. Overcoming such barriers is needed to unlock the full potential of genome editing against nutritional disorders around the world [107]. 

Banana presents an even greater challenge due to its triploid and parthenocarpic nature. Kaur et al. 2020 pioneered the use of CRISPR/Cas9 to alter the carotenoid pathway in banana fruit. Edited lines exhibited up to a sixfold increase in carotene content, accompanied by metabolic reprogramming that significantly reduced lutein and beta-carotene levels. Importantly, these nutritional improvements did not negatively impact agronomic traits, marking the first successful attempt to nutritionally enhance bananas through precise genome editing. Together, these studies illustrate the potential of CRISPR for biofortification, though the method remains technically demanding, especially for crops with complex genomes [108]. 

#### Nanotechnology-enabled biofortification

**Fig. 4:**
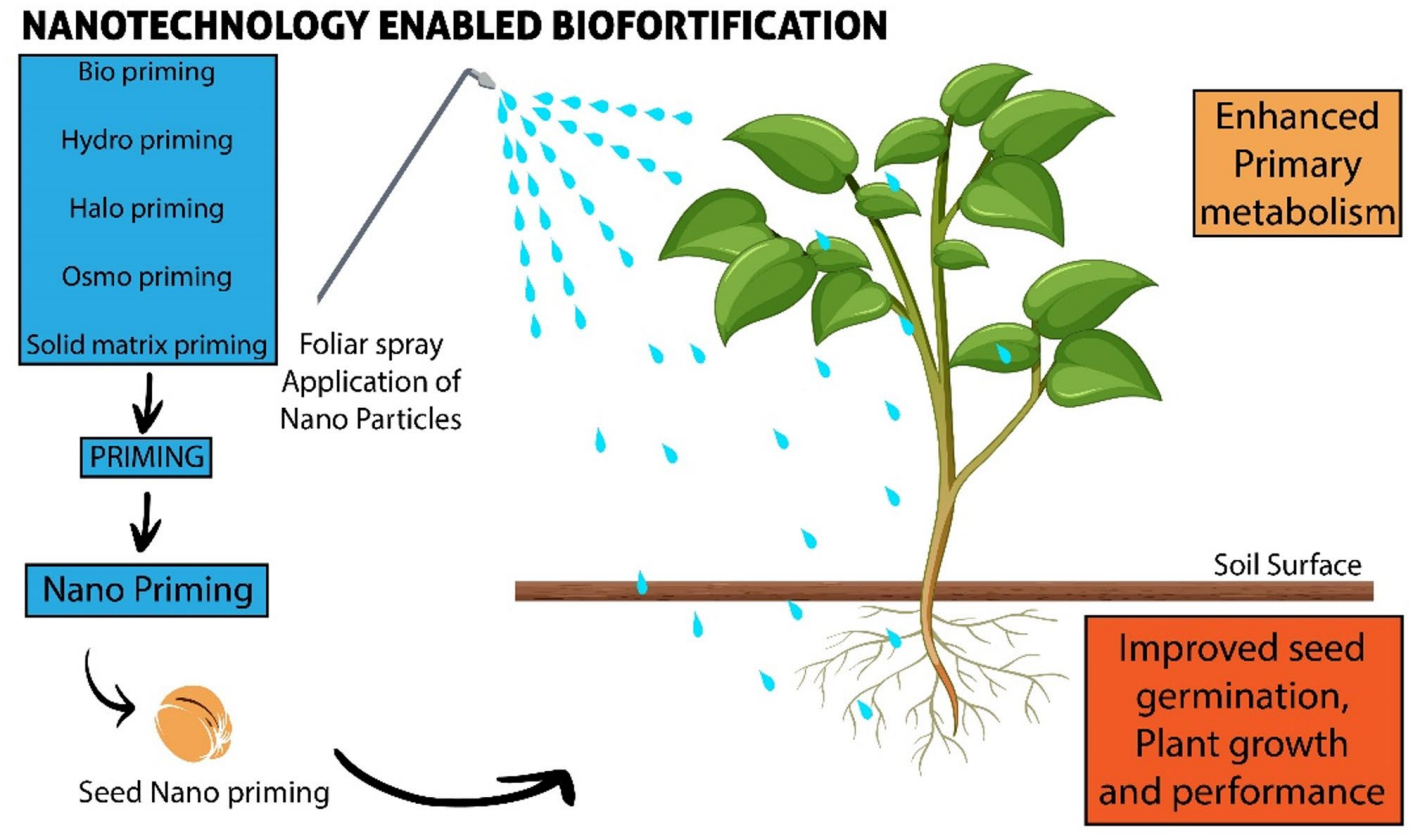
Nanoparticles enabled biofortification

Despite the promise of genetic approaches, challenges such as regulatory hurdles, high cost, and technical limitations necessitate alternative strategies. Agronomic biofortification, though simple, often suffers from inconsistent nutrient uptake due to soil variability and plant physiology [96]. nanotechnology has emerged as a frontier solution to overcome these limitations. By designing nanoscale nutrient carriers, it is possible to enhance nutrient solubility, targeted delivery, and controlled release in plant systems. This approach not only improves uptake efficiency but also minimizes nutrient losses, offering a complementary strategy to conventional and genetic methods of biofortification [109] (Fig. 4).

Conventional chemical fertilizers can provide essential nutrients to plants, but their efficiency is often low, and excessive use can harm both human health and the environment [110]. Nanotechnology offers a promising alternative. Nano-fertilizers, due to their high surface area and controlled particle size, can improve nutrient solubility, uptake efficiency, and translocation within plants, while reducing losses from leaching or soil fixation [111]. Evidence for these benefits is accumulating across multiple crops. In wheat, nanofertilizers have been shown to enhance iron, zinc, and selenium bioavailability, providing a sustainable strategy to address micronutrient deficiencies in staple foods [112]. 

In alfalfa (*Medicago sativa*), Cota-Ruiz et al. [113] demonstrated that nano-Cu treatments improved agronomic performance, enhanced Fe and Zn accumulation, and upregulated stress-responsive enzymes such as superoxide dismutase, suggesting that nanomaterials can influence plant physiology at both molecular and metabolic levels. Similarly, Guha et al. [114] reported that zero-valent iron (nZVI) seed priming in rice improved grain nutrient profiles and yield compared to conventional hydropriming, supporting the use of nanomaterials as “pro-fertilizers” for crop improvement. Beyond primary nutrient delivery, Thakur et al. (2018) and others have highlighted the broader role of nanotechnology in food systems, from nutrient enhancement to reducing post-harvest losses.

Collectively, these studies underscore the advantages of nanotechnology-enabled biofortification: targeted nutrient delivery, higher bioavailability, stress mitigation, and adaptability to soil constraints. However, concerns remain. At high concentrations, nanomaterials may induce phytotoxicity and raise environmental risks [111]. Moreover, large-scale deployment will require optimized application protocols, rigorous safety assessments, and clear regulatory frameworks to ensure responsible use [110]. Khan et al. showed the efficacy of nanoscale interventions in increasing zinc and iron levels in wheat grains, providing a concrete solution to fight iron and zinc deficiencies among staple-dependent populations [112]. 

In a similar vein, Cota-Ruiz et al. [113] investigated the application of copper nanowires in alfalfa and reported positive molecular and physiological responses, indicating a possible application of nanomaterials in enhancing crop nutrient profiles via precision agriculture. Thakur et al. (2018) highlighted the wider application of nanotechnology in food systems, including its application in improving the nutritional value of food products and minimizing post-harvest losses [109]. 

Overall, the research as a whole indicates that nanotechnology-based biofortification has significant promise to alleviate malnutrition, especially in micronutrient-scarce areas. However, its actualization will hinge on reconciling effectiveness with safety, together with regulatory and public education measures for enabling responsible adoption.

Biofortification is a long-term intervention to address micronutrient hidden hunger that operates by increasing the content of key micronutrients such as iron, zinc, and vitamin A in hundreds of staple crops before harvest. The impact of biofortified foods has been found to enhance nutrition among individuals in rural and poor communities who are at risk using long-term and affordable interventions. To extend its reach to more communities and make the world’s nutrition security more robust, it will be crucial to disseminate the technique to more crops, integrate it with emerging technology, and enlist more people in its use.

## Constraints for different biofortification techniques

### Agronomic techniques

Agronomic biofortification, primarily through micronutrient-enriched fertilizers, is an effective but variable strategy for boosting the nutrient content of crops. Its success depends on mineral mobility, plant uptake, and soil composition differ across species and locations. While temporary increases in crop micronutrient levels as possible, this approach often leads to nutrient accumulation in non-edible plant parts, reduced bioavailability due to antinutrients, and demands continuous fertilizer application, water, and labor inputs. Moreover, nutrients may accumulate in non-edible plant parts, such as foliage, rather than in consumable grains or fruits. The presence of antinutrients like phytic acid further reduces the bioavailability of minerals [96]. 

A major challenge lies in environmental sustainability. Excess fertilizer application does not always translate into higher nutrient accumulation in edible plant parts; instead, it can result in nutrient leaching and runoff. For example, phosphate and nitrate fertilizers often enter water bodies, contributing to eutrophication, a process that depletes oxygen and disrupts aquatic ecosystems. Similarly, excessive zinc or iron fertilizers may accumulate in soils, leading to toxicity for soil microbes and reduced biodiversity. Long-term reliance on mineral fertilizers can also lead to soil acidification and structural degradation, ultimately reducing the land’s productivity over time. In India, studies have shown that indiscriminate fertilizer use has increased nitrate concentrations in groundwater beyond safe limits in several agricultural regions, posing risks to both human health and the environment [101]. Agronomic techniques are also highly vulnerable to climatic fluctuations, which can affect nutrient delivery and crop yields. Importantly, since these practices do not alter crop genetics, they cannot address traits such as pest or drought resistance. These limitations suggest that while agronomic techniques are valuable, their effectiveness is maximized when integrated with genetic and biotechnological interventions.

Therefore, while agronomic biofortification remains valuable, especially for rapid nutrition improvements, its greatest efficacy and sustainability arise when combined with genetic and biotechnological enhancements that improve nutrient allocation to edible parts and crop adaptability.

### Traditional breeding techniques

Conventional plant breeding has provided many successful biofortified cultivars and remains a sustainable, long-term, and cost-effective approach. Yet, several constraints hinder its broader application. Progress is inherently slow, often requiring many generations to fix traits, and the approach lacks precision since breeders rely on phenotypic selection rather than direct manipulation of genes. Environmental factors may obscure genetic potential, making trait selection less reliable [96]. Another significant limitation is the restricted genetic variability within crop gene pools. Repeated use of narrow genetic resources risks creating bottlenecks, which not only slow the development of biofortified crops but also increase susceptibility to pests, diseases, and environmental stress. These issues underscore the necessity of integrating traditional breeding with molecular tools and genomic technologies to accelerate progress and enhance resilience [101]. 

New genomic technologies make conventional breeding secondary because they make genetic improvement more precise, quicker, and more efficient. Breeders can identify and modify particular genes or quantitative trait loci (QTLs) associated with desirable micronutrient traits through technologies such as marker-assisted selection (MAS), genome-wide association studies (GWAS), genomic selection, and gene editing (e.g., CRISPR/Cas9). The accuracy is not based on the slow, phenotype-based selection that occurs in many conventional methods. This implies that the environment is less likely to impact the expression of the trait [106]. 

Genomic technologies also help to broaden the genetic basis by making it easier to add useful alleles from different or wild germplasm. This gets rid of problems that come from having small gene pools and makes plants less likely to get pests and stress. New sequencing and “omics” technology help us learn more about the genetic variety of crops that aren’t grown very often. This speeds up the process of finding genes that are linked to nutrition. Genome editing can also target antinutrients to make nutrients more available. The combination of genomic technologies and traditional breeding speeds up the creation of biofortified varieties that have more nutrients and are better able to fight off pests and diseases. These types of plants address both agricultural and nutritional needs in a way that is more efficient and long-lasting [105]. 

### Transgenic techniques

Transgenic biofortification circumvents many limitations of conventional breeding by introducing genes from diverse sources, enabling precise improvements in nutrient content. However, this approach faces some of the strongest constraints. Public resistance remains the greatest challenge, arising from concerns about food safety, environmental risks, ethical issues, and widespread misinformation. Limited transparency in regulatory processes further erodes public trust [96]. Another drawback is that many nations have developed regulatory frameworks for accepting and selling these transgenic crops. Unfortunately, today’s political and economic milieu does not support this technology. Due to the length of time needed from target trait and gene discovery, modification, expression, and characterization of agronomical features to understanding the potential impact on other life forms, the success rate of the transgenic-based strategy in terms of cultivar release is relatively low.

In addition, the regulatory environment for genetically modified organisms (GMOs) is complex, costly, and time-intensive, often delaying commercialization and limiting adoption. Political and economic factors also constrain acceptance across many countries. On the technical side, not all plant species are amenable to genetic transformation, and unintended genetic interactions can sometimes affect plant performance or development. The long pipeline, from identifying target traits to validating field performance, results in a relatively low success rate for cultivar release. Despite these barriers, transgenic techniques remain powerful, but their future depends on strengthening safety assessments, transparent communication, and public engagement to foster broader acceptance [115]. 

Because of concerns for safety, environmental impacts, and ethical challenges, transgenic biofortification techniques have been confronted with a great deal of public resistance and stringent government hurdles. But alongside these societal and governmental issues, technical issues have also made it difficult for them to be applied more extensively in the past. Among the problems are the challenge to alter recalcitrant crop species, the possibility of accidental genetic changes, and the requirement that inserted genes be expressed equally. The latest gene editing technologies, particularly CRISPR/Cas systems, offer us hope that we can address these technical issues. CRISPR allows you to make targeted changes without the introduction of new DNA sequences. This reduces the likelihood of unforeseen effects and accelerates the process of transformation, particularly in recalcitrant crops [108]. 

This innovation not only corrects technical issues, but it may also facilitate easier regulation by allowing scientists to produce cisgenic or altered forms that appear to originate from random mutations. Therefore, the use of gene editing and transgenic technology together is a favorable approach towards addressing technological as well as regulatory issues, making genetically modified biofortified crops more likely to be accepted by society [104]. 

## Biofortified staples in India and its neighboring countries

### Biofortification of rice

Rice (*Oryza sativa*) is the principal staple for over half of the world’s population, yet polished rice consumed widely across South Asia is chronically low in micronutrients such as zinc (Zn) and iron (Fe), contributing to “hidden hunger”. Biofortification, unlike post-harvest fortification, enhances nutrient density directly in crops, making it more suitable for rural communities with limited access to industrially fortified foods [116]. 

In India, several Zn and protein-enriched rice cultivars have been released through conventional breeding. DRR Dhan 49 (Zn-biofortified, ICAR-IIRR) and Zinco Rice MS (IGKV, 27.4 ppm Zn) have been widely tested in multi-location trials [117]. Similarly, CR Dhan 311 and CR Dhan 315 (NRRI, Odisha) show enhanced protein levels (10.2%). Iron-enriched varieties such as IR68144-3b-2-2-3 and Jalmagna reach up to 21 mg/kg Fe [118]. Few rice varieties have undergone rigorous human or community-level evaluation. For instance, DRR Dhan 49 showed increased grain Zn, yet trials on children’s plasma zinc status reported negligible improvements [119].

BRRI dhan 64 and BRRI dhan 72 (Bangladesh) and Zhengdao 11 (China) are nutritionally superior, but evidence of population-level health benefits is still emerging. This highlights the need for long-term clinical validation alongside agronomic performance [120]. 

Biofortification of Chinese rice targets the fortification of rice with micronutrients such as Fe, Zn, and Vitamin A through plant breeding and agronomic methods. The process is intended to enhance the nutritional content of Chinese staple rice and combat micronutrient deficiencies [82]. Initiatives such as HarvestPlus-China, involving local and foreign scientists, have been instrumental in the development and dissemination of biofortified rice varieties. China has the challenges of Fe, Zn, and Vitamin A deficiencies, thereby making biofortification an imperative intervention [121]. Genetic modification and cross-breeding to develop rice varieties with increased micronutrient content. Increasing nutrient absorption by plants through soil and foliar application of fertilizers, including Fe and Zn [122]. 

Indian zinc-biofortified rice lines like DRR Dhan 49 and CR Dhan 311 have been able to achieve high zinc concentrations—up to 26 ppm in milled grains—without adverse effects on yield characteristics. The varieties may provide consumers with 38–57% of the recommended daily allowance (RDA) of zinc. Research using in vitro systems and animal studies verifies that zinc from these biofortified varieties is absorbed almost twice as well as that from well-known non-biofortified varieties, for example, IR64. Early clinical findings from the Indian Council of Medical Research show improvements in plasma zinc status in children eating zinc-biofortified rice; however, large human trials are still required. Further, zinc deposition also differs among soils and agroclimatic regions, showing the necessity for more efficacy data to be specific to settings. These constraints highlight the urgency for additional clinical studies to determine health effects and enable wider utilization (Table. 2).

**Table 2:** Biofortification techniques and crop varieties: key attributes and health evidence

S. No.	Techniques/Variety	Key Advantages	Key Disadvantages	Country/Origin	Target nutrients	Methods	Health Impact Evidence	References
1	Agronomic techniques	Rapid, cost-effective, soil nutrition improvement	Environmental risks, repeated inputs, variable uptake	N/A	Multiple minerals	Fertilizer application	Limited long-term impact	[82, 123]
2	Transgenic techniques	High nutrient levels, novel traits, targeted accumulation	Regulatory burden, public resistance, and ethical concerns	N/A	Multiple micronutrients	Genetic modification	Yet to be widely validated	[124, 125]
3	Traditional breeding	Sustainable, cost-effective, adaptable to local contexts	Slow progress, genetic bottlenecks, labor-intensive	N/A	Multiple nutrients	Crossbreeding, selection	Proven for many varieties	[126, 127]
4	DRR Dhan 49 (Rice)	High zinc content, good yield	Limited clinical trials	India (ICAR-IIRR)	Zinc	Backcross and pedigree selection	Promising plasma zinc data in children	[128]
5	Zinco Rice MS	Zinc enriched	Pending health impact data	India (IGKV)	Zinc	Pure line selection	Not established	[129]
6	CR Dhan 311 (Rice)	Enhanced protein content	Lack of impact studies	India (ICAR-NRRi)	Protein	Backcross and pedigree	No clinical data available	[130]
7	IR68144-3B-2-2-3 (Rice)	Iron biofortified	Limited data on health outcomes	Philippines (IRRI)	Iron	Pedigree selection	Limited	[131]
8	Zincol-2016 (wheat)	Zinc enhanced	Pending widespread validation	Pakistan (NARC)	Zinc	Molecular breeding	Not documented	[132]
9	Black-grained wheat	Zinc, anthocyanin biofortification	Limited health data	India (ICAR-IARI)	Zinc, anthocyanin	Conventional breeding	Not evaluated	[133]
10	HI 1633 (wheat)	Iron, zinc, protein	Pending impact studies	India (ICAR-IIWBR, JNKVV)	Iron, zinc, protein	Conventional breeding	Not available	[134]

Bangladesh mainly has a Zinc deficiency, which can be solved by biofortified Zinc rice. Consuming this rice variety did not affect plasma zinc concentration [126]. Rice varieties BRRI dhan 62, BRRI dhan 72, BRRI dhan 64, and Binadhan-20 produced high Zinc and Iron content in biofortified rice. Conventional breeding has cultivated these, leading to micro deficiency in Bangladesh [135]. 

In Sri Lanka, the biofortification of rice in this country is based on fortified kernel supply to the milling industry, providing simplicity in the blending process and low investment. Pakistan mainly fortified the seeds concerning the climate and location regarding foliar treatment with four micronutrients (Zinc, Iron, Iodine, and Selenium) and, in return, increased the efficiency of that micronutrient deficiency in the crops. In Pakistan, places like Gujranwala-I & II, Sheikhupura-I & II, Sialkot, and Sahiwal–Super Basmati are grown [136]. As a tool for biofortifying Vitamin A rice, Nepal uses the Golden rice aspect to diminish micronutrient deficiency. Still, various issues are prevailing over Golden rice in Nepal. However, it can be accepted nearby [137]. In China, in places like Jiangsu and Chongqing, the biofortified rice varieties Zhengdao 11 and Xiyou 19 are grown, respectively [136]. 

### Biofortification of wheat

Countries suffering mainly from iron deficiency use wheat as their staple food. These countries use biofortified wheat rich in micronutrients to combat malnutrition [138]. Over the past 20 years, significant efforts have been made to increase the biofortification and bioavailability of wheat grain for micronutrients like Se, Fe, and Zn. To improve bioavailability while breeding for micronutrients, gene pools should be examined for the presence of not only the grain, as mentioned above, micronutrients, but also phytic acid (PA), phytate, and phytase. These QTLs/genes and associated markers were found using GWAS and QTL interval mapping, which were then used for marker-assisted selection (MAS) in breeding for biofortification [139]. Factors influencing adoption include seed availability, yield performance, awareness, and market demand, all of which warrant further study to realize biofortification’s potential benefits.

To turn the “Indian thali into a nutri-thali, wheat varieties high in protein, iron, and zinc were also released. The emphasis on biofortification is a step forward in India’s transformation from food availability and access to nutrition security and eradicating hidden hunger. To address anemia, the administration also plans to make rice biofortification mandatory during the following three years. With targeted micronutrients, biofortified wheat is produced with different names such as Zinc Shakti (Chitra), BHU 18, BHU 17, BHU 6, BHU 5, BHU 3, and BHU 1 [140]. 

Zinc Shakti (Chitra), BHU 18, BHU 17, BHU 6, BHU 5, BHU 3, and BHU 1 were produced by conventional methods, and the targeted nutrient level was 40–45 ppm by CIAT, CIMMYT. HD 3171 and PBW 757 were made by a hybridization and selection process with a nutrient level of 47.1 ppm and 42.3 ppm, respectively, by IARI, PAU, India. Pusa Tejas (HI 8759) (durum) and MACS 4028 (durum) have target nutrient levels of 42.1 ppm Iron, 42.8 ppm Zinc, and 12% protein produced by pure line selection [134]. Variety WB2 has a target nutrient level of 40 ppm Iron and 42 ppm Zinc obtained by refined extraction by IIWBR [118]. 

PBW 752 with an efficient target protein level of 12.5% by the conventional method produced as a biofortified protein-rich wheat PAU. HI, 8627 with 6–9 ppm nutrient content produced by conventional breeding in India by IARI [118]. Wheat varieties like NABIMG-9, NABIMG-10, and NABIMG-11 are produced by the backcross method to be rich in biofortified anthocyanin wheat [133]. 

The National Seed Board (NSB) of Bangladesh recently released a new, high-yielding, blast-resistant wheat variety as the nation’s wheat farmers work to recover from an outbreak of the mysterious disease known as “wheat blast” in 2016, according to a communication from the Wheat Research Centre (WRC) in Bangladesh. The International Wheat Improvement Network, which turns 50 this year, shared the conventional cross-breeding technique used to develop the wheat line utilized in BARI Gom 33 with Bangladesh and other South Asian co-operators [141]. 

The continuous threat of wheat blast disease in Bangladesh has a direct impact on the stability of wheat production and food security. To ensure that crops with enhanced nutrients are able to cope with biotic issues, it is necessary to work on developing wheat varieties that are both biofortified and blast-resistant. This joint emphasis on enhancing nutrition and disease resistance in crops will be extremely crucial to sustained acceptability and having an impact in blast-prone regions. This indicates why it will be necessary to incorporate disease resistance into programs for developing biofortified wheat.

Due to the lack of economic analysis, foliar Zn application for biofortification has only been tested on a small scale. In order to measure the health burden, China’s Ministry of Health performed the first cost-effectiveness analysis using the “disability-adjusted life year” method. In three major wheat-growing regions of China, the cost of agronomic biofortification with Zn of wheat was thus calculated [142]. Anthocyanin biofortified wheat was produced in China from black-grained wheat with a nutrient target level of 17.71% prtein, produced by conventional breeding.

In 2016, Pakistan saw the release of Zincol-2016, a new strain of wheat biofortified with zinc. This study’s main objective was to investigate how Zincol-2016 wheat flour affected biochemical and functional indicators of zinc status in a population with a pervasive zinc deficiency. By conventional breeding, Zincol 2016, NR 419, 421 aimed to increase the nutrient level by 33.9 ppm, produced in cooperation with CIMMYT [132]. 

Between genotypes, there were differences in the grain’s Zn and Fe concentrations of 25.8 to 42.4 ppm and 20.6 to 57.9 ppm, respectively. Genotype 7HPYT409 had the highest grain zinc concentration (42.4 ppm), followed by genotype 7HPYT410 (39.6 ppm) [143]. With 3821 kg/ha, genotype 7HPYT448 produced the most grain, followed by 7HPYT418 (3760 kg/ha), 7HPYT426 (3688 kg/ha), and 7HPYT413 (3592 kg/ha). The genotype 8HPYT417 had the highest grain zinc concentration (37.5 ppm), followed by genotypes 8HPYT404 (34.4 ppm) and 8HPYT443 (33.5 ppm). ((PDF) Evaluation of Biofortified Spring Wheat Genotypes for the Terai Region of Nepal, 2017) Similar results were seen for grain yield in IET-biofortified 2017/18 (p 0.05). With a grain yield of 3365 kg/ha, genotype NL 1387 leads the pack, followed by NL 1389 (3288 kg/ha), NL 1379 (3030 kg/ha), NL 1385 (2994 kg/ha), and NL 1345 (2864 kg/ha) [143]. Table 2 illustrates the commonly used biofortified wheat varieties in different countries.

### Other crops

Apart from rice and wheat, some biofortified crops are also being developed and promoted in India and other neighboring countries to fight micronutrient deficiency. Pearl millet has been biofortified in India with improved iron and zinc content. Cultivars such as ‘Dhanashakti’ and hybrids such as ‘AHB 1200 Fe’ and ‘HHB 299’ have established notable increases in micronutrient levels and yield. For example, ‘Dhanashakti’ holds 71 mg/kg of iron and produces approximately 2.2 t/ha, while newer hybrids hold iron between 83–91 mg/kg and zinc between 39–46 mg/kg with over 3 t/ha yields. These millets have been incorporated into public health initiatives to tackle anemia and zinc deficiencies. Lentils are also given prominence, with types such as ‘Pusa Ageti Masoor’ and ‘IPL 220’ bred to have greater iron and zinc content. ‘Pusa Ageti Masoor’ contains 65 ppm iron and takes 100 days to mature, being appropriate for rainfed conditions, while ‘IPL 220’ has 73 ppm iron and 51 ppm zinc and takes 121 days to mature [144]. 

Millets and lentils often have lower input requirements, greater resilience to climate variability, and higher micronutrient densities—particularly in iron, zinc, and protein—making them cost-effective candidates for biofortification. Additionally, millets’ shorter growing cycles and lentils’ nitrogen-fixing ability contribute to agroecological sustainability and lower production costs. However, their limited cultivation area and lower consumer demand compared to rice and wheat present challenges in scaling biofortification impact.

In Bangladesh, orange-fleshed sweet potato (OFSP) with vitamin A has been introduced to reduce vitamin A deficiency. Likewise, Pakistan has been promoting biofortified maize with provitamin A and zinc wheat to address micronutrient malnutrition. These are components of larger efforts to diversify diets and enhance nutritional outcomes in the region. Together, these biofortified crops present environmentally sustainable alternatives to micronutrient deficiency, particularly where such staples form a crucial part of the diet [145]. 

By comparing factors such as breeding complexity, required investments, time to market, and nutritional payoff across these crops, the narrative can better inform policy and investment decisions, showing where biofortification efforts can yield the highest return in health and food security outcomes. This would also highlight opportunities for diversifying staple food systems to improve dietary quality and resilience.

## Government schemes and policies to strengthen biofortification strategies

Government efforts at scaling up biofortification reveal compelling success and clear structural barriers across regions. More serious and structured examination of existing policies and schemes is enhanced by aggregation according to intervention type as well as regional setting, emphasizing policy design, incentives, and implementation issues from India, South Asia, and industrialized nations.

### India: subsidies, programs, and integration

The Indian government guides biofortification via a variety of schemes: ICAR’s NARI scheme, “Nutri-smart” villages, Frontline Demonstrations of biofortified crops, and convergence with welfare platforms such as the Mid-Day Meal, Anganwadi, and National Food Security Mission. State governments (Odisha, Bihar) directly finance biofortified seed production/distribution, frequently using local institutions and livelihood agencies to facilitate diffusion among poor rural groups. Incentives also encompass subsidized seed, technical assistance, and pilot “nutritional villages,” which enhance market entry for smallholder farmers [145]. 

### South Asia (Bangladesh, Nepal): policy, procurement, and scale-up

Strong government procurement (social safety nets), public-private stakeholder partnership, and widespread awareness drives mainstreaming of zinc-biofortified rice by Bangladesh, supported by partners such as GAIN [146]. 

Nepal, with international research centers (CIMMYT), launched biofortified wheat through fast-track breeding, focusing on regional crop adaptation and solid government seed dissemination systems [147]. 

### Developed countries (US, UK): research networks and innovation platforms

The UK and US focus on advanced R&D (e.g., vitamin D-enriched tomatoes, B12-fortified peas) and international science collaborations for improved agricultural biofortification platforms.

Support takes the form of bioscience network funding (Biofortification Hubs), regulatory systems, and industry-academic partnerships [148]. 

### Structural analysis: policy strategies and implementation issues

Policy instruments comprise direct government investment in R&D, policy requirements for participation in public food programs, subsidized input supply, and regulatory incentives like tax relief [149]. 

Public-Private Partnerships (PPP): Important for scaling and diffusion to the market, particularly in South Asia, where private input sellers, seed firms, and NGOs engage in breeding, testing, and marketing of biofortified varieties. Implementation difficulties are significant: low farmer knowledge, irregular market demand, shortcomings in supply chains, and the requirement for durable political will and consumer education, especially in low-income and rural environments [150]. Though POSHAN Abhiyaan labeled millets as “Nutri Cereals” in acknowledgement of their nutritional significance, the initiative has not succeeded in enhancing the utilization of biofortified millets in food systems as yet. This shortage is due to a series of ongoing gaps, some of which are operational, incentive-based, and structural in nature [118]. 

#### Gaps in coordination and structural policy

In spite of the policy focus on millets, POSHAN Abhiyaan and its associated programs (NFSM, RKVY) have focused more on calorie supplementation and fortification than on using biofortified millets in existing procurement and meal schemes for the vulnerable segments. Nutrition objectives have not been uniformly translated into agriculture procurement priorities by virtue of inadequate cross-ministerial coordination (MWCD, MoHFW, and Agriculture) [151]. Last-mile delivery issues, such as poor tracking of which Anganwadi centers or PDS outlets are actually dispensing biofortified foods, are another issue that POSHAN Abhiyaan suffers from. This leads to low coverage and minimal impact on nutrition indicators [152]. 

#### Operational and market barriers

Lack of adequate infrastructure, distribution channels, and seed multiplication has prevented biofortified millets, particularly pearl millet, from having supply and farmer adoption challenges persist. The ability to reliably supply biofortified crop produce is limited by infrastructure deficiencies, such as irrigation and storage in semi-arid millet-growing regions. This is especially true since hybrid biofortified seed may be less tolerant or less palatable to farmers than conventional open-pollinated varieties. Taste, health benefit ignorance, and low consumer demand also discourage the market’s uptake of biofortified millets, which reduces financial incentives for farmers and food distributors [146]. 

#### Programmatic and awareness issues

Since the majority of nutrition communications are oriented toward calorie consumption or overall diet diversity and not toward specific micronutrient-enriched staple crop varieties, POSHAN Abhiyaan’s promotion of biofortification remains low. Even in pilot schemes with organizations such as Akshaya Patra, extension services and local procurement organizations very rarely push or give priority to biofortified millet in public distribution systems, school meals, and supplementary nutrition programs [153]. 

These public schemes and policies, if carried out in an integrated and multi-faceted way, play a pivotal role in unlocking the full potential of biofortification in combating malnutrition and bringing about food and nutrition security. The emphasis lies in establishing an enabling environment that favors the cultivation, dissemination, and utilization of nutrient-rich crops [151]. 

## Conclusion

Malnutrition remains a critical challenge in South Asia, including India, Pakistan, Bangladesh, Nepal, Sri Lanka, and China, where micronutrient deficiencies in iron, zinc, and vitamin A disproportionately affect children, adolescent girls, pregnant women, and the elderly. While economic progress has improved food availability, nutritional security lags behind, demanding targeted solutions. Biofortification offers a powerful tool to enhance the nutritional quality of staple foods, but its real potential lies in tailoring interventions to demographic needs and embedding them within existing policy frameworks. Linking biofortified crops with school feeding programs, government nutrition missions, and public distribution systems can maximize reach and impact.

Biofortification processes need to transition beyond patchy pilots to integrated policy and market incorporation. The integration of CRISPR and nanotechnology within breeding schemes and the creation of demographic-targeted delivery, for example, Zn-wheat for older citizens, are essential next steps. Challenging the imperative of transgenic R&D in the face of effective agronomic approaches calls for policy reorientation and budget reallocation. An integrated South Asian task force on biofortification, following the example of Bangladesh’s zinc rice achievement, can facilitate harmonized regional scale-up. Systematic and progressive changes are imperative to make long-term impacts on nutrition.

Emerging innovations, such as CRISPR-mediated biofortification, nanotechnology-based nutrient delivery, and microbe-assisted agronomic approaches, must now transition from research pipelines to national deployment. To avoid fragmented progress, a coordinated South Asian biofortification task force should be established, drawing lessons from successful examples like Bangladesh’s zinc rice program, which achieved large-scale adoption. Moreover, research priorities should balance short-term agronomic interventions with long-term genetic solutions, ensuring ecological sustainability and cost-effectiveness.

Going forward, biofortification must evolve from isolated pilot projects into an integrated regional strategy that combines crop improvement, nutrient policy, and public health delivery. Greater investment in farmer training, consumer awareness campaigns, and decentralized seed systems will be crucial to accelerate adoption. Strengthening public-private partnerships can further drive innovation, scale, and affordability of biofortified crops. Finally, rigorous monitoring and impact assessment frameworks should be built into all interventions to ensure that biofortification translates into measurable improvements in nutritional outcomes. With coordinated action between governments, research institutions, and stakeholders, South Asia can move decisively toward eradicating hidden hunger and building resilient, healthier populations.

## Data Availability

The dataset generated and/or analyzed during the current study is available from the corresponding author (Dr. Sandeep Singh Rana), upon reasonable request.
